# Electroacupuncture inhibits weight gain in diet-induced obese rats by activating hypothalamicLKB1-AMPK signaling

**DOI:** 10.1186/s12906-015-0667-7

**Published:** 2015-05-12

**Authors:** Jing Xu, Liang Chen, Lewei Tang, Le Chang, Si Liu, Jinfeng Tan, Yinglong Chen, Yulan Ren, Fanrong Liang, Jin Cui

**Affiliations:** Chengdu University of Traditional Chinese Medicine, 610075 Chengdu, Sichuan China; Guiyang College of Traditional Chinese Medicine, No. 50 Shi Dong Road Guiyang Province, 550002 Guiyang, Guizhou China; The Second Affiliated Hospital and Yuying Children’s Hospital of Wenzhou Medical University, 325027 Wenzhou, China

**Keywords:** Obesity, Electroacupuncture, AMPK, LKB1, ACC

## Abstract

**Background:**

Electroacupuncture (EA) is reported to be an effective treatment for obesity, but its mechanism is unclear. This study aimed to investigate the relationship between hypothalamic LKB1-AMPK-ACC signaling and EA.

**Methods:**

Fifty male Sprague–Dawley rats were divided into two groups fed either chow (chow-fed group) or high-fat diet (HF group). After 4 weeks of feeding, obese rats in the HF group (defined as weighing 20 % or more than rats in the chow-fed group) were randomly allocated into an EA or Diet-induced obesity (DIO) group. The EA group was given EA on bilateral ST25–ST36 for 4 weeks, while the DIO group received no further intervention. Body weight of the chow-fed, DIO, and EA groups were measured weekly. mRNA and protein levels of the hypothalamic LKB1-AMPK-ACC signaling pathway were detected using real-time (RT)-PCR and western blot, respectively.

**Results:**

After 4 weeks of EA treatment, the weight growth trend of rats in the EA group was inhibited compared with those in the DIO group. RT-PCR and western blotting showed that EA upregulated the transcription of Adenosine 5′-monophosphate -activated protein kinase α2 (AMPKα2), promoted protein expression of Liver kinase B1 (LKB1) and AMPKα1, and inhibited acetyl-CoA carboxylase (ACC) protein expression in the hypothalamus.

**Conclusions:**

This study suggests that hypothalamic LKB1-AMPK-ACC signaling plays an important role in EA treatment for obesity.

## Background

Obesity is a worldwide public health problem, and can lead to diseases like coronary heart disease, diabetes, and some cancers [1]. Several investigations into curbing the increasing obese population have been conducted, but there are few effective pharmacological treatments. Only three drugs have been approved by the US Food and Drug Administration for long-term obesity treatment, namely lorcaserin (Belviq), phentermine plus topiramate (Qsymia), and orlistat (Xenical, Alli) [2].

Acupuncture is one of the most popular alternative therapies, and it has been used to treat obesity for thousands of years. Electroacupuncture (EA) is a common form of acupuncture in which an electric current is passed through acupuncture needles. The parameters of the EA can be precisely characterized, so it is reproducible, and data suggest that EA may be more effective than manual acupuncture [3]. Recent systematic reviews indicate that EA is an effective treatment for obesity [4]. However, the mechanism of EA on obesity needs further investigation. There is evidence indicating that EA may suppress appetite to control weight. The possible mechanisms underlying the effect of EA focus on the hypothalamus [5, 6].

The hypothalamus regulates food intake and energy balance. Hypothalamic adenosine 5′ monophosphate-activated protein kinase (AMPK) is recognized as a nutrient and glucose sensor in the central nervous system, and a regulator of appetite [7, 8]. Liver kinase B1 (LKB1) is the major upstream kinase in the AMPK cascade. LKB1 is constitutively active and phosphorylates AMPK at Thr172 of the α subunit [9–11]. AMPK activation is abolished in cells lacking LKB1 expression or in rodents after deletion of LKB1 [12, 13]. One mechanism by which AMPK regulates lipid metabolism is via phosphorylation and inactivation of acetyl-CoA carboxylase (ACC), an important rate-limiting enzyme for the synthesis of malonyl-CoA [14]. ACC is both a precursor for fatty acid biosynthesis and an inhibitor of long-chain fatty acyl-CoA transport to mitochondria for β-oxidation [15]. Knockdown/knockout of ACC1 and ACC2 was reported to cause continuous fatty acid oxidation, increased energy expenditure, and reduced fat mass [16, 17].

Several studies have studied the relationship between EA and AMPK. Tominaga et al. [18] suggested that repeated EA therapy is capable of improving diet-induced insulin resistance, possibly via AMPK signaling activation in skeletal muscle. Immediately after EA stimulation, phospho-AMPKα (Thr172) was significantly higher in animals receiving EA than control animals. Kim et al. [19] showed that levels of AMPK gene expression in the rat hypothalamus determine individual differences in the sensitivity to EA-induced analgesia. However, there has been no thorough investigation into EA and AMPK in the hypothalamus.

Based on a diet-induced obesity (DIO) rat model, we observed whether EA can inhibit weight gain in rats given an HF diet. We also investigated the role of hypothalamic LKB1-AMPK-ACC signaling in the obesity pathology and EA treatment mechanism.

## Methods

### Animals and experimental protocol

54-week-old male Sprague–Dawley rats (80–100 g) were obtained from Dossy Experimental Animals Company (Chengdu, China). Animals were housed in a facility with ambient temperature (22 ± 2 °C) and maintained in 12/12 h light–dark cycles (light on from 07:00 to 19:00). To acclimatize to the new environment, all rats were fed with standard laboratory chow and water available *ad libitum* during the first week of the experiment. Animals were randomly divided into two groups: a chow-fed group and a high-fat (HF) group. The chow-fed group (n = 13) was given standard laboratory chow (Dossy Experimental Animals Company, 3.80 kcal/g), composed of 5 % fat, 55 % carbohydrates, 22 % protein, 7 % ash, and 5 % fiber. The HF group (n = 35) was given an HF diet (4.72 kcal/g) composed of 22 % fat, 39 % carbohydrate, 23.7 % protein, 4 % ash, and 3 % fiber. HF food was made in the authors’ laboratory. Each 100 g of HF food was composed of basic feed (57.5 g), egg yolk powder (11.79 g), lard (10 g), pig bile salt (0.2 g), casein (7 g), milk power (13 g), salt (0.085 g), and yeast powder (0.425 g). Body weight was monitored once every week at 09:00.

After feeding for 4 weeks, 24 rats fed the HF diet had gained weight 20 % or more above the average weights of rats in the chow-fed group, and were defined as obese. These rats were then randomly allocated into an EA group receiving EA stimulation for four weeks, and a DIO group receiving no further treatment, with 12 rats in each group. The EA, DIO, and chow-fed groups were housed individually, and fed with their corresponding diets. Food intake and body weight were measured daily for 4 weeks. Hypothalami were collected at the end of the study. The study was approved by the Institutional Animal Care and Use Committee of Chengdu University of Traditional Chinese Medicine and all procedures were conducted in accordance with Animal Experiments Guidelines and Animal Care of Chinese Academy of Sciences.

### EA treatment

Rats in the three groups were consciously restrained in a plastic holder. The EA group was treated with EA a bilateral Tianshu (ST25) and Zusanli (ST36) acupoints for 20 min from 8:00 to 12:00 a.m., once a day, 6 days per week, for four weeks in total. The needles used were disposable sterile stainless steel needles with diameter 0.30 mm and length 25 mm (Suzhou Hua Tuo Medical Instruments Co. Ltd, Suzhou, China). Points were chosen based on the standards for rats, as recorded in Experimental Acupuncture and Moxibustion [20]. Tianshu (ST25) is located 5 mm lateral to the navel (5 mm lateral to the intersection between the upper 2/3 and the lower 1/3 in the line between xiphoid process and pubic symphysis upper border). Zusanli (ST36) is located between the tibia and fibula at approximately 5 mm lateral and 5 mm lower to the anterior tubercle of the tibia. The acupoints were stimulated with a continuous-wave electrical stimulus with an intensity of 2 mA and frequency of 3 Hz for 20 min to produce slight twitches in the limbs. The G6805-II EA instrument (No. 20,101,014, Qingdao Xinsheng Ltd., Qingdao, China) was used. Rats in the chow-fed and DIO groups were restrained for 20 min without EA stimulation. The rats did not show any pain or discomfort during the treatments.

All experimental rats were fasted for 10 h after the last intervention was given, and their body weight was recorded (g). Under 20 % urethane (0.8 g/kg) anesthesia, rats were euthanized. Then, hypothalamic tissues were dissected for further tests.

### Western blot analysis

Hypothalami were dissected using the optic chiasm as a rostral landmark, and the mammillary bodies caudally to a depth of 2 mm. Dissected hypothalami were immediately frozen in liquid nitrogen. Tissues were homogenized in ice-cold lysis buffer containing 0.1 % SDS, 10 mM Tris–HCl (pH 7.4), 1 % Triton X-100, 1 mM MgCl_2_, and 1 % NP-40. Homogenates were centrifuged at 10,000 *g* for 10 min at 4 °C, supernatants were removed, and aliquots were snap-frozen in liquid nitrogen. Hypothalamus lysate (40 μl) was subjected to sodium dodecyl sulfate - polyacrylamide gel electrophoresis (SDS-PAGE) on 6 % polyacrylamide gels and electrotransferred on a nitrocellulose membrane (Millipore, Massachusetts, USA).

Membranes were blocked for 1.5 h in bovine lacto transfer technique optimizer hybridization solution (50 mM Tris [pH 8.0], 2 mM CaCl_2_, 0.01 % Antifoam A, 0.02 % NaN_3_, and 0.05 % Tween 20) containing 5 % skim milk. Membranes were then probed at 4 °C in Tris-buffered Saline and Tween 20 (TBST) overnight with the appropriate dilution of the indicated antibodies against LKB1 (Abcam, Cambridge, UK), ACC (Abcam, Cambridge, UK), p-AMPKα1 (Abcam, Cambridge, UK), AMPKα1 (Saierbio, Tianjin, China), p-AMPKα2 (Abcam, Cambridge, UK), AMPKα2 (Saierbio, Tianjin, China), and GAPDH (Saierbio, Tianjin, China).

Detection of proteins was performed using horseradish peroxidase—conjugated secondary antibodies (goat anti-rabbit antibody, Saierbio, Tianjin, China) and an enhanced chemiluminescence reagent (Western Lightning-ECL; Perkin Elmer, Waltham, MA, USA), then exposed to film. The intensity of protein bands was quantitated using Lab Works 4.0 software (UVP Inc., Upland, CA, USA).

### RNA extraction and real-time PCR

Rat tissue was isolated, frozen in liquid nitrogen, and stored at −80 °C until extraction. Total RNA was extracted from about 100 mg of hypothalamus using TRIzol (Invitrogen, Carlsbad, CA, USA), as described by the manufacturer. The purity and concentration of isolated RNA was determined by measuring absorbance at 260 and 280 nm using a Biophotometer (Eppendorf, Hamburg, Germany). An aliquot (5 μg) of extracted RNA was reverse transcribed into first-strain complementary DNA (cDNA) using an M-MLV RT reagent (Promega, Madison, WI, USA). The following thermal cycling protocol was used for reverse transcription: 42 °C for 30 min, and 70 °C for 10 min. Real-time PCR was performed with a Bio-Rad iQ5 thermal cycler (Bio-Rad Laboratories, Hercules, CA, USA) using SYBR Premix Ex Taq™ (Takara, Dalian, China). Thermocycling was carried out in a final volume of 20 μl containing 0.5 μl of the cDNA sample, 10 μl of SYBR Green Mix (Takara), and 2 μl of primers (forward and reverse primers, 5 pmol/μl). The PCR protocol included initial denaturation of 4 min at 94 °C, followed by 40 cycles of amplification for 30 s at 94 °C, 30 s at 50 °C, and 40 s at 72 °C. Triplicate samples were run for real-time PCR. Relative expression was calculated as follows: density of the product of respective target gene divided by that for GAPDH from the same cDNA. Specific primers used for PCR are listed in Table 1.

**Table 1:** Primers used in real-time PCR

Gene	Primer sequence	Gene number	Product length
LKB1 F	GGCTCTTGTGCCTATCCC	NM001108069.1	201 bp
LKB1 R	CCATTCTGACCCACTTCCTC	NM001108069.1	201 bp
ACC F	CGGCAACAAACAAGGG	NM053922.1	260 bp
ACC R	CGTTACAACCAGGAAGCC	NM053922.1	260 bp
AMPKα1 F	CCTACCACCTCATAATAGACAA	NM019142.2	348 bp
AMPKα1 R	GGTTCTTCCTTCGCACA	NM019142.2	348 bp
AMPKα2 F	CTGAGAAGCAGAAGCACGAC	NM023991.1	537 bp
AMPKα2 R	CTGCATAATTTGGCGATCC	NM023991.1	537 bp
GAPDH F	CAGTGCCAGCCTCGTCTCAT	NM017008.4	595 bp
GAPDH R	AGGGGCCATCCACAGTCTTC	NM017008.4	595 bp

### Statistical analysis

All experimental data are expressed as means ± standard deviations. Statistical analysis was performed by SPSS Statistics for Windows, Version 20.0 (IBM, Armonk, NY, USA) and one-way ANOVA for comparisons among groups. For all analyses, P < 0.05 was considered to be statistically significant.

## Results

### EA inhibits weight gain from an HF diet

**Fig. 1:**
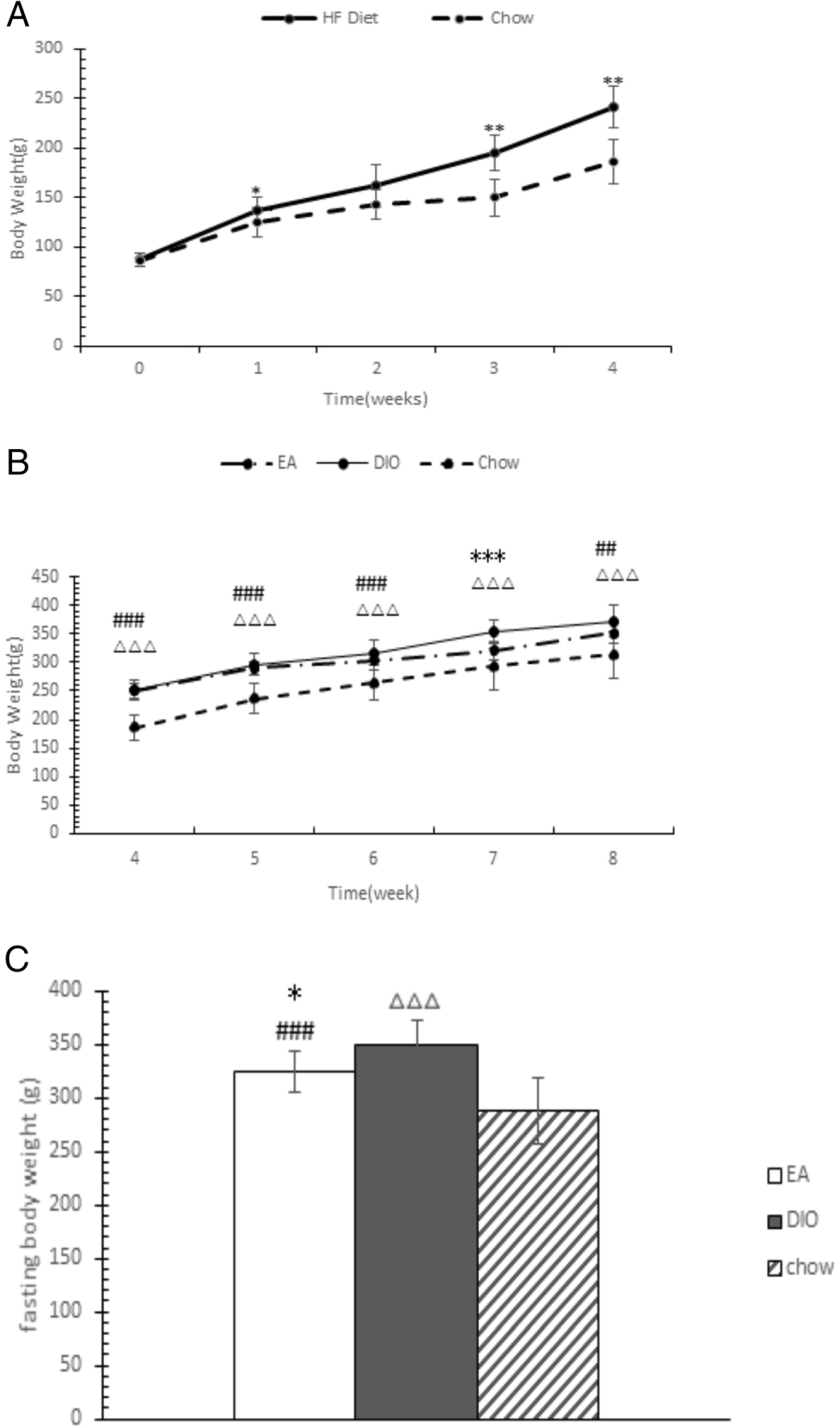
Body weight changes before and after diet-induced obesity model establishment. (**a**) Body weight gain in high fat (HF) and chow-fed rats. (**b**) Body weight gain in EA, diet-induced obesity (DIO), and chow-fed rats. (**c**) Body weight after 10-h fasting of electroacupuncture (EA), DIO, and chow-fed rats. *P < 0.05, **P < 0.01, ***P < 0.001 EA vs. DIO; ^#^P < 0.05, ^##^P < 0.01, ^###^P < 0.001, EA vs. chow; ^∆^P < 0.05, ^∆∆^P < 0.01, ^∆∆∆^P < 0.001 DIO vs. chow

Figure 1a shows that rats in the HF-fed group gained more body weight than those in the chow-fed over 4 weeks. Differences in body weight were noticeable from 1 week, but were significant after 4 weeks. 24 rats fed the HF diet became obese (gained 20 % or more weight above the average weight of rats in the chow-fed group), and were separated into two groups at the end of 4 weeks. There were 12 rats in the DIO group, and 12 in the EA group.

Figure 1b shows that EA treatment inhibits body weight gain in rats fed an HF diet. The most significant differences between the EA and DIO groups were observed in the seventh week (P = 0.001), although the body weights between the two groups were not significantly different (5.49 % lower in the EA group, P = 0.129) at the end of the experiment. Figure 1c shows that rats in the EA group had a significantly lower body weight compared with those in the DIO group after fasting for 10 h (P = 0.027).

### Effects of EA on hypothalamic AMPK signaling

**Fig. 2:**
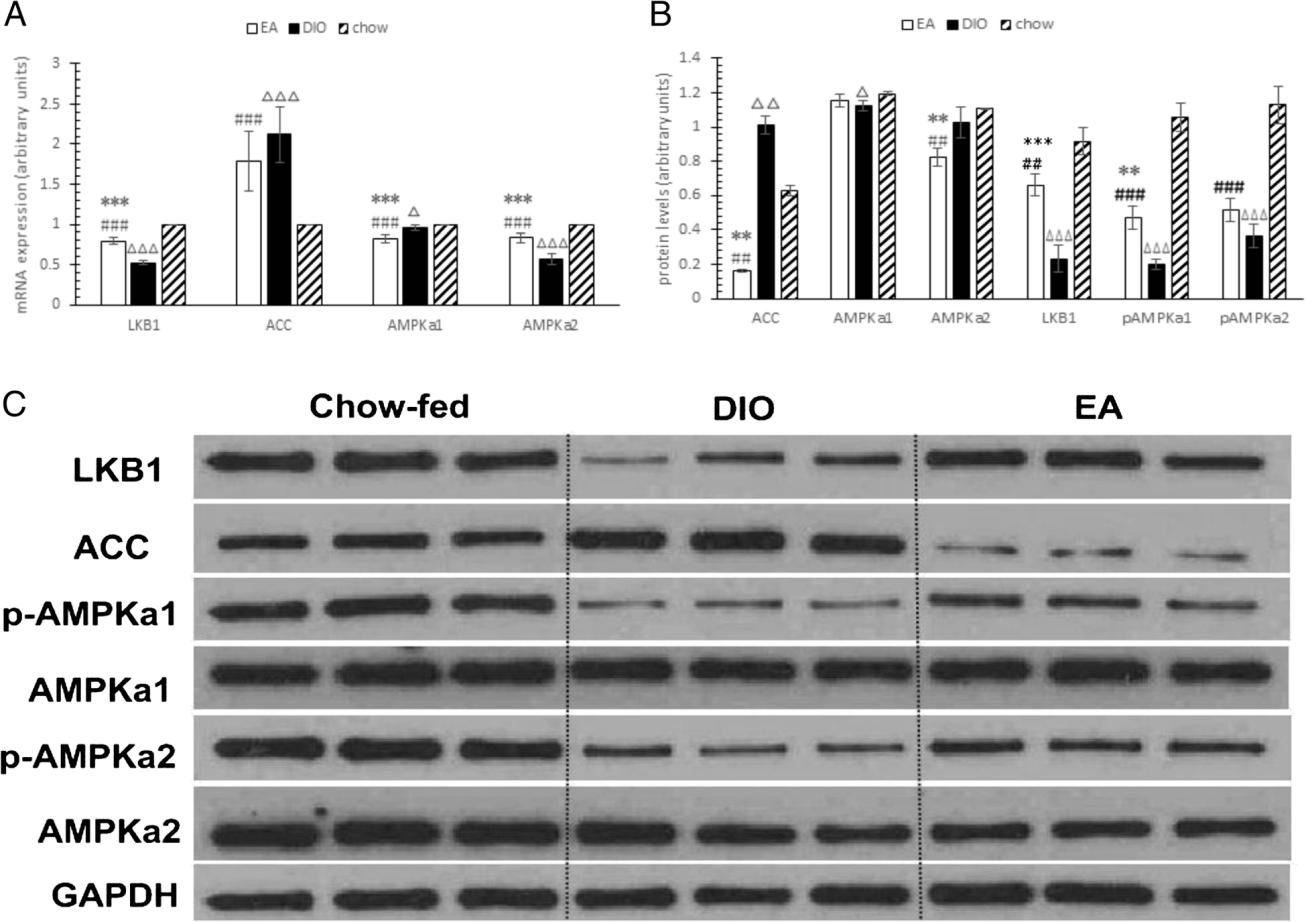
Effects of electroacupuncture on adenosine 5′-monophosphate-activated protein kinase signaling in hypothalamus. (**a**) mRNA levels of liver kinase B1 (LKB1), acetyl-CoA carboxylase (ACC), Adenosine 5′-monophosphate -activated protein kinase (AMPK)α1, and AMPKα2 in each group. (**b**) Quantization of protein levels in each blot by densitometry shown in (**c**). (**c**) Representative western blots in each group to show LKB1, ACC, AMPKα1, and AMPKα2 protein content. *P < 0.05, **P < 0.01, ***P < 0.001 electroacupuncture (EA) vs. diet-induced obesity (DIO); ^#^P < 0.05, ^##^P < 0.01, ^###^P < 0.001, EA vs. chow; ^∆^P < 0.05, ^∆∆^P < 0.01, ^∆∆∆^P < 0.001 DIO vs. chow

Figure 2a shows that rats fed an HF diet had lower expression of LKB1 (P = 0.000), AMPKα1 (P = 0.020), and AMPKα2 (P = 0.000), and significantly higher transcription of ACC (P = 0.024) than rats in the chow-fed group. The EA group had approximately 48.03 % more AMPKα2 (P = 0.000) and 13.59 % less AMPKα1 mRNA (P = 0.000) transcripts than that in the DIO group.

Figure 2b shows that HF diet can suppress the protein contents of LKB1 (P = 0.000), AMPKα1 (P = 0.019), pAMPKα1 (P = 0.000), and pAMPKα2 (P = 0.000), and increase the content of ACC (P = 0.005). The EA group had 182.89 % higher LKB1 (P = 0.000), 135.42 % higher pAMPKα1 (P = 0.002), and 84.27 % lower ACC (P = 0.003), 19.72 % lower AMPKα2 (P = 0.007) protein levels than those in the DIO group.

## Discussion

We observed that after 4 weeks of HF diet, nearly 50 % of rats were obese. During the 4-week EA treatment, rats in the EA group gained weight slower than those in the DIO group starting in the third week. RT-PCR and western blotting showed that LKB1-AMPK signaling in the hypothalamus is inhibited by HF diet, and ACC is significantly upregulated. EA may upregulate transcription of AMPKα2, promote protein expression of LKB1 and AMPKα1, and inhibit ACC protein expression to control weight gain.

AMPK is widely expressed in neurons and astrocytes of the hypothalamus and hindbrain, which are both areas involved in food intake [21]. Under normal physiological conditions, hypothalamic AMPK can be activated by infusing 5-aminoimidazole-4-carboxamide 1-β-D-ribofuranoside (AICAR) into the third ventricle, which significantly increases food intake [22]. Expressing dominant negative AMPK in the hypothalamus can reduce food intake and body weight [23].

Feeding mice a HF diet will cause dysregulation of AMPK signaling pathway, which is associated with impaired AMPK phosphorylation and downregulated protein expression in skeletal muscle, liver, and hypothalamus [24–27].

Under pathological conditions, a strong correlation between low activation state of AMPK and metabolic disorders such as obesity, insulin resistance, and sedentary activities has been established in a variety of rodent models [28, 29]. Therefore, mice lacking AMPK might be more sensitive to the deleterious effects of over-nutrition [30]. Consistent with this hypothesis, whole-body ablation of AMPKα2 activity exacerbates HF diet-induced obesity, while the glucose disposal rates are similar to those of wild-type mice [31].

As one of the most important regulators of energy balance, AMPK has close relationship with many metabolic-related hormones, especially adipocytokines, such as leptin, adiponectin and apelin. In physiology circumstance, leptin [32] exerts an inhibitory effect on AMPK in the hypothalamus by stimulating ACC and subsequently suppressing food intake, meanwhile constitutive activation of hypothalamic AMPK disrupts leptin’s anorexigenic effect. In addition, inhibition of hypothalamic ACC attenuates leptin-mediated inhibition in food intake and body weight gain [33]. Latterly, it was shown that mTOR/S6K regulates eating through leptin- mediated inhibition of AMPK in the hypothalamus [34]. AMPK [35] signaling also regulated adiponectin production by modulating the expression of its receptors and itself. Apelin [36] is an adipocytokine known for its anti-obesity and anti-diabetic properties, Apelin promotes the expression of anti-oxidant enzymes and suppresses the expression of pro-oxidant enzyme via AMPK pathway.

Therefore, AMPK has emerged as a promising new target for the treatment of metabolic disorders including obesity, type 2 diabetes, and cardiovascular disease. Activation of AMPK using AICAR can increase glucose uptake and fatty acid oxidation in obese diabetic rodents [37, 38] and humans [39–43], which validates the therapeutic potential of an AMPK activator.

pAMPK is the activated state of AMPK, which is phosphorylated on threonine residue 172 (Thr-172) in the α subunit by upstream kinases like LKB1 [44]. The combination of the allosteric and phosphorylation effects causes a greater than 1000-fold increase in kinase activity (compared with less than 5-fold for allosteric activation alone). This response allows for high sensitivity in responses to small changes in cellular energy status [45]. Activated AMPK catalyzes the dephosphorylation of ACC, then increases the level of hypothalamic malonyl-CoA which results in food intake suppression and an eventual energy expenditure increase [46].

Our results demonstrate that EA rats had significantly higher protein levels of LKB1 and pAMPKα1 compared with DIO rats. Therefore, EA may promote the activity of hypothalamic AMPK by increasing its phosphorylation level, and this effect may induced by an increase in its upstream kinase, LKB1. Meanwhile, the decrease in ACC protein level may be the result of AMPK activation. Alterations in the hypothalamic LKB1-AMPK-ACC signaling pathway might contribute to the effect of EA on slowing down weight gain in rats given an HF diet. However this hypothesis should be investigated further, possibly with hypothalamic LKB1 and AMPK knockdown rat models.

Intriguingly, we found significant weight differences between the EA and DIO groups after 3 weeks and after 10 h fasting on the eighth week, but not on the eighth week immediately after EA treatment. This is possibly because acupuncture increases basal metabolic rate (BMR) in the obese state [47]. SMR is the steady-state rate of heat production by a whole organism under a set of standard conditions. It is either measured directly as heat production or indirectly as oxygen consumption. The mainly contribution of the cellular processes that underlie SMR includes a futile cycle of proton extrusion across the mitochondrial inner membrane and the subsequent proton leak back to the matrix via endogenous proton conductance pathways, which accounted for about one-half of the oxygen consumption rate of resting [48]. The proton leak is catalyzed by uncoupling proteins (UCPs), UCP1, UCP2, and UCP3, and the classic uncoupling protein is UCP1, which uncouples brown adipose tissue (BAT) mitochondria causing facultative thermogenesis [49]. When fully activated, UCP1 in BAT can increase metabolic rate in rodents by fourfold [50]. Peroxisome proliferator-activated receptor gamma coactivator 1α (PGC-1α) is a crucial activator of UCP1, which induces the expression of the genes encoding for UCP1 [51]. Increasing PGC-1α and UCP1 expressions are considered to be treatment targets of obesity and obesity-related diseases [52]. It is reported that EA can upregulate PGC-1α mRNA (2–3-fold) to ameliorate insulin resistance in obese and diabetic db/db mice [53]. Du H [54] found that EA effectively induces the mRNA expression of PGC-1α and UCP-1 by 4-fold and 5-fold in the BAT of obese rats, respectively. Shen WX [55] found that UCP1 gene expression in white adipose tissue (WAT) increased almost 14 folds in EA mice than in the control mice, and the UCP1 protein expression also significantly increased in EA mice. They presumed that EA can remodel WAT to BAT by inducing UCP-1 expression, and this may be one of the mechanisms by which acupuncture affects weight loss. Noteworthily, AMPK can activates PGC-1α by phosphorylating on its specific serine and threonine residues [56]. So EA may increase BMR through AMPK - PGC-1α - UCP-1 pathway. Furthermore, EA was found to increase small intestinal transit. Acceleration of intestinal transit was found to be associated with reduced absorption of nutrients [57]. Therefore, the accelerative effect of EA on intestinal transit might contribute to reduced body weight [58]. Our body weight result is also confirmed by other research [59]. It was reported that body mass reduction was about two-fold greater in control versus high-fat mice, possibly because fasting-induced responses occur sooner in obese animals, especially decreases in VO2, heat production, and nocturnal activity. Because of above reasons, 10 h fasting enlarged weight differences between EA and DIO group, then made it statistically significant. This phenomenon may reflect sustainable effect of EA treatment for obese and deserve further research.

We chose ST36 and ST25 as acupoints in this study for a few reasons. Firstly, previous study showed that EA treatment on these acupoints significantly reduced food intake and body weight [60, 61]. Secondly, these two acupoints are the most commonly used by others to treat obesity either in animal studies or in clinical studies [62]. Third, stimulation of these acupoints was reported to increase AMPK expression in some tissues [18, 19]. However, a few reports showed that EA ST36 may enhanced appetite to a certain extent [63], but more studies suggested that EA ST36 significantly inhibited feeding and body weight gain [64–69]. The possible mechanisms are down-regulated orexigenic peptides, such as neuropeptide Y (NPY) [64], upregulates anorexigenic hormones, including proopiomelanocortin (POMC) [65], α-Melanocyte-stimulating hormone (α-MSH) [66], and cholecystokinin (CCK) [67–69]. Nevertheless, there are other acupoints used for the treatment of obesity, further studies should test whether stimulation of other acupoints acts through a similar mechanism like stimulation of ST36 and ST25.

## Conclusions

Dysregulation of hypothalamic LKB1-AMPK-ACC signaling was detected in DIO rats. EA treatment can inhibit weight gain in DIO rats fed an HF diet. RT-PCR and western blotting showed that EA may act via upregulation of AMPKα2 transcription, promotion of LKB1 and AMPKα1 protein expression, and inhibition of ACC protein expression to control energy balance. This study suggests that hypothalamic LKB1-AMPK-ACC signaling plays an important role in EA treatment for obesity.

## Abbreviations

ACC: Acetyl-CoA carboxylase
AICAR: 5-aminoimidazole-4-carboxamide 1-β-D-ribofuranoside
AMPK: Adenosine 5′-monophosphate -activated protein kinase
α-MSH: α-Melanocyte-stimulating hormone
BMR: Basal metabolic rate
BAT: Brown adipose tissue
CCK: Cholecystokinin
DIO: Diet-induced obesity
EA: Electroacupuncture
GAPDH: Glyceraldehyde phosphate dehydrogenase
HF: High fat
LKB1: Liver kinase B1
NPY: Neuropeptide Y
PGC-1α: Peroxisome proliferator-activated receptor gamma coactivator 1
POMC: Proopiomelanocortin
RT-PCR: Real-time polymerase chain reaction
SDS-PAGE: Sodium dodecyl sulfate-polyacrylamide gel electrophoresis
TBST: Tris-buffered saline and tween 20
UCP-1: Uncoupling protein-1
